# Hemagglutinin Stalk Antibody Responses Following Trivalent Inactivated Influenza Vaccine Immunization of Pregnant Women and Association With Protection From Influenza Virus Illness

**DOI:** 10.1093/cid/ciz927

**Published:** 2019-09-27

**Authors:** Nisha Dhar, Gaurav Kwatra, Marta C Nunes, Clare Cutland, Alane Izu, Raffael Nachbagauer, Florian Krammer, Shabir A Madhi

**Affiliations:** 1 Medical Research Council, Respiratory and Meningeal Pathogens Research Unit, Faculty of Health Sciences, University of the Witwatersrand, Johannesburg, South Africa; 2 Department of Science and Technology, National Research Foundation, Vaccine Preventable Diseases, Faculty of Health Sciences, University of the Witwatersrand, Johannesburg, South Africa; 3 Department of Microbiology, Icahn School of Medicine at Mount Sinai, New York, USA

**Keywords:** influenza, immunization, pregnant, stalk antibody, protection

## Abstract

**Background:**

The conserved, immuno-subdominant influenza virus hemagglutinin (HA) stalk region is a potential universal group-specific influenza virus vaccine epitope. We analyzed antibody responses to H1 hemagglutinin stalk domain (H1/stalk) following trivalent influenza inactivated vaccine (IIV3) immunization in pregnant women, and association with protection against influenza virus illness.

**Methods:**

One hundred forty-five human immunodeficiency virus (HIV)–uninfected pregnant women (68 IIV3 and 77 placebo recipients) and 140 pregnant women with HIV infection (72 IIV3 and 68 placebo recipients) were independently randomized in placebo-controlled efficacy trials of IIV3. Plasma samples were tested for H1/stalk immunoglobulin G (IgG) and hemagglutination inhibition (HAI) antibodies prevaccination and 1 month postvaccination. Women had weekly surveillance for influenza illness, confirmed by polymerase chain reaction.

**Results:**

Increases in H1/stalk IgG (and HAI) antibody levels were elicited post-IIV3, with responses being higher in HIV-uninfected women than in women living with HIV. Among HIV-uninfected vaccinees, there was no correlation (postvaccination) between H1/stalk and HAI antibody responses, whereas a strong correlation was observed in vaccinees with HIV. The H1/stalk IgG concentration was lower among women developing A/H1N1 illness (85.3 arbitrary units [AU]/mL) than those without A/H1N1 illness (219.6 AU/mL; *P* = .001). H1/stalk IgG concentration ≥215 AU/mL was associated with 90% lower odds (odds ratio, 0.09; *P* = .005) of A/H1N1 illness. Also, H1/stalk IgG was significantly lower among women with influenza B illness (93.9 AU/mL) than among their counterparts (215.5 AU/mL) (*P* = .04); however, no association was observed after adjusting for HAI titers.

**Conclusions:**

H1/stalk IgG concentration was associated with lower odds for A/H1N1 influenza virus illness, indicating its potential as an epitope for a universal vaccine against group 1 influenza virus.

## Teaser

This study showed an association of H1 hemagglutinin stalk-specific immunoglobulin G in broader protection from influenza virus infections in pregnant women in a placebo-controlled trial of an inactivated influenza vaccine trial. The findings have implications on designing more-effective influenza vaccine candidates.

The effectiveness of current seasonal influenza virus vaccines is unpredictable, including low or no effectiveness when there is antigenic mismatch between the seasonal vaccine and circulating strains [1]. Immunogenicity of inactivated influenza vaccines (IIVs) is usually analyzed using hemagglutination inhibition (HAI) assay, which measures antibodies targeted against the globular head domain of hemagglutinin (HA), the immunodominant major surface glycoprotein of the virus [2–6]. The focus on HAI immune responses in predicting IIV effectiveness is predicated upon studies from the 1960s that demonstrated that strain-specific HAI titers ≥1:40 were associated with 50% reduced risk of influenza illness in healthy adults [7, 8]. The HA head domain, however, exhibits high antigenic plasticity. This results in antigenic drift that enables evasion from HA antibody responses induced by previous exposure [9].

We previously reported, in 2 separate randomized controlled trials in people living with human immunodeficiency virus (HIV), of the disconnect between trivalent IIV (IIV3)–induced HAI titers and the vaccine efficacy observed against confirmed influenza illness [10, 11]. In both studies, humoral responses to HA were low to modest in adults living with HIV, with 35%–43% [11] to 53%–71% [10] of vaccinees having seroconverted to the different vaccine strains postvaccination. In contrast, in HIV-uninfected women, 65%–92% of vaccinees had seroconverted postvaccination [11]. Nevertheless, the vaccine efficacy point estimate against polymerase chain reaction (PCR)–confirmed influenza illness was higher in women living with HIV (70.6% [95% confidence interval {CI}, 23.0%–88.8%]) than in HIV-uninfected women (54.4% [95% CI, 19.5%–74.2%]) [11]. Similarly, high vaccine efficacy was also observed in an earlier randomized controlled trial in adults living with HIV (75.5% [95% CI, 9.2%–95.6%]) [10, 11]. These findings suggest that IIV, especially in people with HIV infection, induces immune responses other than HAI responses that likely contribute to protection against influenza illness.

Efforts are under way to identify conserved influenza virus epitopes as targets for broadly protective or universal influenza virus vaccines [12, 13]. This includes a focus on the HA stalk domain, which is immuno-subdominant compared to the globular head, but antigenically more conserved. The HA stalk domain is largely conserved within influenza A (group 1 and group 2) and influenza B viruses [14, 15] and undergoes limited antigenic changes [16, 17]. Stalk-specific antibodies are broadly neutralizing in vitro, and protect against influenza virus challenge in murine and ferret models against multiple subtypes of the same HA group [12, 15, 18–21].

The objective of this study was to investigate immunoglobulin G (IgG) response to the H1 hemagglutinin stalk domain (H1/stalk) following IIV immunization in pregnant women with or without HIV infection, and evaluate the association of H1/stalk IgG and odds for developing influenza virus illness.

## METHODS

### Study Design

We retrospectively tested plasma samples from women enrolled as part of an immunogenicity study in 2 parallel randomized, placebo-controlled trials on IIV3 in pregnant women with or without HIV infection, as described previously [11]. In brief, the participants, stratified by HIV status, were randomly assigned in a 1:1 ratio to receive IIV3 or placebo. Eligibility criteria included an age of 18–38 years and an estimated gestation of 20–36 weeks. Influenza virus vaccine (Vaxigrip) contained 15 µg each of A/California/7/2009 (A/H1N1/pdm09), A/Victoria/210/2009 (A/H3N2), and a B/Brisbane/60/2008-like virus (B/Victoria) as recommended by the World Health Organization for the Southern Hemisphere in 2011 and 2012. The women with HIV were enrolled in an immunogenicity study in 2011, whereas the HIV-uninfected women (188 IIV3 vaccinees and 188 placebo recipients) were enrolled as a nested immunogenicity cohort in a phase 3 efficacy trial in 2011 and 2012. The immune responses measured by HAI titers in these women has been reported previously [11]. In this study, analyses was limited to participants enrolled in 2011 in whom postvaccination samples were collected within 16–42 days (median, 30 days) postimmunization (Supplementary Table 1). Plasma samples archived at −70°C from the 2011 immunogenicity cohort including 140 IIV3 vaccinees (72 with HIV and 68 HIV-uninfected) and 145 placebo recipients (68 with HIV and 77 HIV-uninfected) were tested for H1/stalk domain IgG prior to and after randomization at the Respiratory and Meningeal Pathogens Research Unit, University of the Witwatersrand (Johannesburg, South Africa). The women underwent active surveillance for influenza virus illness during the study and were tested for influenza virus using PCR assay as described previously [11]. Participants in whom PCR-confirmed influenza illness occurred before the post-IIV3 vaccination sampling (in IIV3 recipients) were excluded. All PCR-confirmed influenza cases were stratified for group 1 (A/H1N1) and non–group 1 virus (ie, A/H3N2 or B/Victoria or B/Yamagata lineages) illnesses. The same participants were analyzed for association between strain-specific HAI titers and risk for influenza illness by the homotypic virus.

### Measurement of H1/Stalk IgG

For detection of H1/stalk IgG, chimeric H6/1 recombinant protein was utilized containing an H6 head domain (to which humans are naive) linked with an H1 stalk domain, to which humans are known to have preexisting immunity [22]. Plasma samples were tested for H1/stalk IgG using an enzyme-linked immunosorbent assay, as detailed in the Supplementary Data. The H1/stalk IgG concentration was quantified using an in-house reference serum composed of purified pooled human IgG (Polygam National Bioproducts Institute, South Africa) with an assigned arbitrary value of 1000 arbitrary units (AU) per milliliter. Plasma samples were tested for HAI titers at the University of Colorado in Denver as described elsewhere [11].

### Data Analysis

We pooled data from participants with HIV and those without HIV to assess the association between H1/stalk antibody concentrations and risk for influenza virus illness, using the 1-month postimmunization levels IgG in IIV3 recipients and the baseline visit IgG concentration in placebo recipients. The baseline and postvaccination antibody concentrations were compared using Wilcoxon matched-pairs signed-rank test for skewed data sets or Student *t* test for data sets in Gaussian distribution. Antibody concentrations between groups were compared using Mann-Whitney test. For all analyses, *P* values, geometric means, and 95% CIs were reported. For categorical variables, groups were compared using Fisher exact test reporting the odds ratio (OR). Reverse cumulative plots were constructed and threshold concentrations were determined.

Logistic regression reporting of the OR was used to identify associations between antibody concentrations and influenza illness with H1/stalk IgG and HAI titers as covariates. The association was further adjusted (reporting adjusted OR [aOR] by HIV status, vaccination status, H1/stalk antibody, and HAI titers as covariates. Data were analyzed using GraphPad Prism version 7.03 software (GraphPad Software, San Diego, California) and Stata version 13 software (StataCorp, College Station, Texas). Correlation analysis was performed using Spearman correlation test. For all analyses, a *P* value <.05 was considered statistically significant.

### Ethical Considerations

The study was approved by the Human Research Ethics Committee of the University of the Witwatersrand (approval numbers 101106 and 101107). All study participants provided written informed consent for inclusion into the parent study.

## RESULTS

Demographic and baseline clinical characteristics were similar between IIV3 and placebo recipients among HIV-uninfected women and women living with HIV, except for mean age being higher among the HIV-infected placebo group (28.8 ± 5.2 years) than the IIV3 group (26.9 ± 4.9 years) (*P* = .02; Supplementary Table 1). There was no difference in characteristics between participants with and without serum samples available for H1/stalk IgG testing except for mean age being higher among participants with nonavailability of serum (29.1 ± 4.5 years) than those whose serum was available for testing (26.9 ± 5.2 years) (*P* = .009; Supplementary Figure 1 and Supplementary Table 2).

### H1/Stalk Antibody Responses to IIV3 Vaccination

Among IIV3 recipients, the H1/stalk IgG geometric mean concentration (GMC) increased 2.24-fold between prevaccination (204.2 AU/mL) and postvaccination (457.9 AU/mL) (*P* < .0001) in HIV-uninfected women; and by 1.79-fold in women living with HIV (116.5 vs 209.3 AU/mL) (*P* < .0001; Table 1 and Supplementary Figure 2). Both prevaccination and postvaccination H1/stalk IgG concentrations were higher in HIV-uninfected IIV3 recipients compared with those living with HIV, as was the fold increase in IgG concentration postvaccination (*P* = .009; Table 1).

**Table 1.** H1/Stalk Immunoglobulin G Responses Among Vaccinated Pregnant Women With or Without Human Immunodeficiency Virus Infection

Response	IIV3 (n = 68)	Placebo (n = 77)	*P* Value
Women living without HIV			
Baseline antibodies, GMC, AU/mL (95% CI)	204.2 (165.8–251.4)	202 (166.1–245.7)	.89^a^
Postvaccination antibodies, GMC, AU/mL (95% CI)	457.9 (393.5–532.7)	233.6 (187.7–290.6)	< .0001^a^
*P* value^b^	< .0001	.98	
Mean fold change (95% CI)	2.24 (1.95–2.57)	1.15 (1.01–1.32)	< .0001^a^
Women living with HIV	IIV3 (n = 72)	Placebo (n = 68)	
Baseline antibodies, GMC, AU/mL (95% CI)	116.5 (96.05–141.3)	104.6 (86.66–126.3)	.42^c^
Postvaccination antibodies, GMC, AU/mL (95% CI)	209.3 (170.5–257)	107.7 (88.71–130.8)	< .0001^c^
*P* value^b^	< .0001	.26	
Mean fold change (95% CI)	1.79 (1.55–2.07)	1.03 (.94–1.12)	< .0001^a^

Comparison of baseline antibody concentration between vaccinees with and those without HIV (*P* = .0001, Student *t* test). Comparison of postvaccination-induced antibody concentration between vaccinees with and those without HIV (*P* < .0001, Mann-Whitney test). Comparison of fold change in antibody concentration between vaccinees with and those without HIV (*P* = .009, Mann-Whitney test).

Abbreviations: AU, arbitrary units; CI, confidence interval; GMC, geometric mean concentration; HIV, human immunodeficiency virus; IIV3, trivalent inactivated influenza vaccine.

^a^Mann-Whitney test.

^b^Wilcoxon matched-pairs signed-rank test.

^c^Student *t* test.

Among placebo recipients, there was no change in H1/stalk IgG concentration 1 month after randomization in either HIV-uninfected participants or participants with HIV. Consequently, H1/stalk IgG was higher among IIV3 than placebo recipients 1 month postrandomization in HIV-uninfected women (457.9 vs 233.6; *P* < .0001) and women living with HIV (209.3 vs 107.7; *P* < .0001) (Table 1 and Supplementary Figure 2).

### Strain-specific HAI Antibody Titers in IIV3 Vaccinees

We previously reported on HAI titers in the HIV-uninfected women (2011 and 2012 cohort) and women living with HIV (2011 only) [11]. In the current study, analysis was limited to the 2011 cohorts. In brief, HAI geometric mean titers (GMTs) 1 month postvaccination were higher among IIV3 than placebo recipients in HIV-uninfected women and women living with HIV (Supplementary Table 3). Furthermore, HAI GMTs 1 month postvaccination were higher in HIV-uninfected IIV3 recipients compared with recipients living with HIV for all 3 strains, and the mean fold increase in HAI titers was also higher in HIV-uninfected women (5.1- to 11.3-fold) than in women living with HIV (2.3- to 3.4-fold) (*P* < .0001 for all strains; Supplementary Table 3).

### Correlations of Antibody Responses Among IIV3 Recipients

Prior to IIV3 vaccination, significant correlation was evident between H1/stalk IgG and A/H1N1 HAI titers in HIV-uninfected women (*r* = 0.51, *P* < .0001) and women living with HIV (*r* = 0.41, *P* = .0003), but not so for H1/stalk IgG vs A/H3N2 or B/Victoria HAI titers (Table 2 and Supplementary Figure 3). One month following IIV3 vaccination, no correlation was observed between H1/stalk IgG and A/H1N1 HAI titers in HIV-uninfected women (*r* = 0.02, *P* = .83), whereas the correlation was stronger in women living with HIV (*r* = 0.71, *P* < .0001) than before vaccination. Also, a significant correlation was observed 1 month following vaccination in women living with HIV who received IIV3 between H1/stalk IgG and A/H3N2 HAI titers (*r* = 0.59, *P* < .0001) and B/Victoria HAI titers (*r* = 0.52, *P* < .0001), as well as a modest correlation for HAI titers to B/Victoria (*r* = 0.37, *P* = .001) in HIV-uninfected IIV3 recipients (Table 2 and Supplementary Figure 3).

**Table 2.** Correlations Between H1/Stalk Immunoglobulin G Concentrations and Hemagglutination Inhibition Titers Before and After Trivalent Inactivated Influenza Vaccination in Women With or Without Human Immunodeficiency Virus

Study Group	H1/Stalk IgG Concentrations vs A/H1N1 HAI Titers, *r* (95% CI)	H1/Stalk IgG Concentrations vs A/H3N2 HAI Titers, *r* (95% CI)	H1/Stalk IgG Concentrations vs B/Victoria HAI Titers, *r* (95% CI)
Pre–IIV3 vaccination			
Women living without HIV (n = 68)	0.51 (.31–.67), *P* < .0001	0.12 (−.12 to .35), *P* = .32	0.27 (.02–.48), *P* = .02
Women living with HIV (n = 72)	0.41 (.19–.59), *P =* .0003	0.23 (.001–.45), *P* = .04	−0.007 (−.24 to .23), *P* = .95
Post–IIV3 vaccination			
Women living without HIV (n = 68)	0.02 (−.22 to .26), *P* = 0.83	0.03 (−.20 to .28), *P* = .75	0.37 (.14–.56), *P* = .001
Women living with HIV (n = 72)	0.71 (.57–.81), *P* < .0001	0.59 (.41–.73), *P* < .0001	0.52 (.33–.68), *P* < .0001

Abbreviations: CI, confidence interval; HAI, hemagglutination inhibition; HIV, human immunodeficiency virus; IgG, immunoglobulin G; IIV3, trivalent inactivated influenza vaccine; *r*, Spearman correlation coefficient.

Among HIV-uninfected IIV3 recipients, the correlation between the prevaccination and 1-month postvaccination H1/stalk IgG concentration was stronger (*r* = 0.69, *P* < .0001) than the corresponding analyses of HAI titers to A/H1N1 (*r* = 0.29, *P* = .01), A/H3N2 (*r* = 0.36, *P* = .002), or B/Victoria (*r* = 0.06, *P* = .59) (Supplementary Table 5 and Supplementary Figure 4). In contrast, among IIV3 recipients with HIV infection, there was strong correlation between the pre- and postvaccination levels for H1/stalk IgG (*r* = 0.76, *P* < .0001) as well as for A/H1N1 (*r* = 0.67, *P* < .0001), A/H3N2 (*r* = 0.70, *P* < .0001), and B/Victoria (*r* = 0.31, *P* = .007) HAI titers (Supplementary Table 5 and Supplementary Figure 4).

### Association of H1/Stalk Antibodies With Influenza Illness

There were 23 confirmed influenza illnesses overall, including 19 (10 A/H1N1, 5 A/H3N2, 1 B/Victoria, and 3 B/Yamagata) in women living with HIV and 4 cases (1 each of A/H1N1, A/H3N2, B/Victoria, and B/Yamagata) in HIV-uninfected women. Overall, H1/stalk IgG GMC was lower among women developing A/H1N1 illness (85.3 AU/mL) than those who did not develop A/H1N1 illness (219.6 AU/mL) (*P* = .001; Table 3 and Supplementary Figure 5). Consequently, in the logistic regression analysis, H1/stalk IgG concentration was associated with lower odds of acquiring A/H1N1 illness (OR, 0.06; *P* = .002), which remained significant after adjusting for HIV status (aOR, 0.12; *P* = .02) or vaccine status (aOR, 0.07; *P* = .004).

**Table 3.** H1/Stalk Immunoglobulin G Concentrations and Association With Confirmed Group 1 A/H1N1 Influenza Illness

All IIV3- or Placebo-Vaccinated Women	A/H1N1 Illness (n = 11)^a^	No A/H1N1 Illness (n = 274)^b^	*P* Value^c^	OR (95% CI); *P* Value^d^	aOR^e^ (95% CI); *P* Value^d^	aOR^f^ (95% CI); *P* Value^d^
H1/stalk IgG GMC, AU/mL (95% CI)	85.3; (48.3–150.6)	219.6; (196.7–245.3)	.001	0.06 (.01–.37); .002	0.12 (.02–.72); .02	0.07 (.01–.43); .004
H1/stalk IgG concentration, AU/mL	Proportion (%)	Proportion (%)	OR (95% CI); *P* Value^g^			
≥50	8/11 (72.7)	258/274 (94.1)	0.16 (.04–.62); .02			
≥100	5/11 (45.4)	217/274 (79.1)	0.21 (.07–.78); .01			
≥150	3/11 (27.2)	183/274 (66.7)	0.18 (.05–.62); .01			
≥200	2/11 (18.1)	149/274 (54.3)	0.18 (.03–.73); .02			
≥215	1/11 (9)	143/274 (52.1)	0.09 (.008–.54); .005			
≥250	1/11 (9)	136/274 (49.6)	0.10 (.009–.60); .01			
≥300	1/11 (9)	110/274 (40.1)	0.14 (.01–.88); .05			
≥350	1/11 (9)	91/274 (33.2)	0.20 (.01–1.19); .11			
≥400	0/11 (0)	74/274 (27)	0 (0–1.0); .07			

At IgG concentrations of ≥215 AU/mL, 90% of women are likely to remain A/H1N1 uninfected as determined from reverse cumulative plot.

Abbreviations: aOR, adjusted odds ratio; AU, arbitrary units; CI, confidence interval; GMC, geometric mean concentration; IgG, immunoglobulin G; IIV3, trivalent inactivated influenza vaccine; OR, odds ratio.

^a^Ten women living with human immunodeficiency virus (HIV); 1 HIV-uninfected woman.

^b^One hundred thirty women living with HIV; 144 HIV-uninfected women.

^c^Mann-Whitney test.

^d^Derived using logistic regression analysis.

^e^Adjusted odds ratio for HIV status using multivariate logistic regression analysis.

^f^Adjusted odds ratio for vaccination status using multivariate logistic regression analysis.

^g^Fisher exact test.

The H1/stalk IgG threshold significantly associated with the lowest odds (OR, 0.09) of developing A/H1N1 illness was ≥215 AU/mL, which was prevalent among 9% and 52.1% (*P* = .005) of women with and without A/H1N1 illness, respectively (Table 3 and Supplementary Figure 5).

H1/stalk IgG GMCs were also lower in women with non–group 1 influenza illness (ie, A/H3N2 or influenza B) (121.4 AU/mL) compared to their counterparts (217 AU/mL) (*P* = .04; Table 4 and Supplementary Figure 6). This difference in H1/stalk IgG GMC between those with and without illness was significant for illness due to influenza B (93.9 vs 215.5 AU/mL; *P* = .04), but not significant for A/H3N2 illness (157 vs 213.1; *P* = .44). In the logistic regression analysis, the H1/stalk IgG concentration was associated with significantly lower odds of influenza B illness (OR, 0.10; *P* = .03), including after adjusting for HIV infection status (aOR, 0.10; *P* = .05). For influenza illness due to non–group 1 viruses, there was a trend of lower odds for acquiring illness with increasing H1/stalk IgG concentration, albeit not significant at any threshold (Table 4 and Supplementary Figure 6).

**Table 4.** H1/Stalk Immunoglobulin G Concentrations and Association With Confirmed Non–Group 1 (A/H3N2 or B) Influenza Illnesses

All IIV3- or Placebo- Vaccinated Women	A/H3N2 or B Illness (n = 12)^a^	No A/H3N2 or B Illness (n = 273)^b^	*P* Value^c^	OR (95% CI); *P* Value^d^	aOR^e^ (95% CI); *P* Value^d^	aOR^f^ (95% CI); *P* Value^d^
H1/stalk IgG, GMC, AU/mL (95% CI)	121.4 (69.6–211.8)	217 (194–242.7)	.04	0.21 (.04–.92); .03	0.29 (.06–1.43); .13	0.34 (.06–1.70); .19
H1/stalk IgG concentration, AU/mL	A/H1N1 Illness, Proportion (%)	No A/H1N1 Illness, Proportion (%)	OR (95% CI); *P* Value^g^			
≥50	11/12 (91.6)	255/273 (93.4)	0.77 (.11–8.81); .57			
≥100	7/12 (58.3)	215/273 (78.7)	0.37 (.12–1.08); .14			
≥150	6/12 (50)	180/273 (65.9)	0.51 (.15–1.69); .35			
≥200	3/12 (25)	148/273 (54.2)	0.28 (.08–1.05); .07			
≥250	3/12 (25)	134/273 (49)	0.34 (.09–1.29); .14			
≥300	3/12 (25)	108/273 (39.5)	0.50 (.14–1.91); .37			
≥330	2/12 (16.6)	96/273 (35.1)	0.36 (.07–1.63); .22			
≥350	1/12 (8)	91/273 (33.3)	0.18 (.01–1.04); .11			
≥400	1/12 (8)	73/273 (26.7)	0.24 (.02–1.43); .19			
≥450	0/12 (0)	59/273 (21.6)	0 (0–1.23); .13			
	A/H3N2 Illness (n = 6)^h^	No A/H3N2 Illness (n = 279)^i^	*P* Value^c^			
H1/stalk IgG, GMC, AU/mL (95% CI)	157 (61.0–403.5)	213.1 (190.7–238.2)	.44			
	B/Victoria or Yamagata Illness (n = 6)^j^	No B/Victoria or Yamagata Illness (n = 279)^k^	*P* Value^c^	OR (95% CI); *P* Value^d^	aOR^e^ (95% CI); *P* Value^d^	aOR^f^ (95% CI); *P* Value^d^
H1/stalk IgG GMC, AU/mL (95% CI)	93.9 (38.5–228.7)	215.5 (193–240.7)	.04	0.10 (.01–.88); .03	0.10 (.01–1.08); .05	NA
H1/stalk IgG concentration, AU/mL	B/Victoria or Yamagata Illness, Proportion (%)	No B/Victoria or Yamagata Illness, Proportion (%)	OR (95% CI); *P* Value^g^			
≥50	5/6 (83.3)	261/279 (93.5)	0.34 (.04–4.27); .34			
≥100	4/6 (66.6)	218/279 (78.10)	0.55 (.12–3.0); .61			
≥150	3/6 (50)	183/279 (65.5)	0.52 (.12–2.28); .42			
≥180	1/6 (16.6)	160/279 (57.3)	0.14 (.01–1.10); .08			
≥200	0/6 (0)	151/279 (54.1)	0 (0–.61); .01			

Abbreviations: aOR, adjusted odds ratio; AU, arbitrary units; B/Victoria or Yamagata, Influenza B/Yamagata strain; CI, confidence interval; GMC, geometric mean concentration; IgG, immunoglobulin G; IIV3, trivalent inactivated influenza vaccine; NA, analysis could not be adjusted for vaccination status as all influenza illness cases were in placebo group; OR, odds ratio.

^a^Nine women living with human immunodeficiency virus (HIV); 3 HIV-uninfected women.

^b^One hundred thirty-one women living with HIV; 142 HIV-uninfected women.

^c^Derived from Mann-Whitney test.

^d^Derived using logistic regression analysis.

^e^Adjusted odds ratio for HIV status using multivariate logistic regression analysis.

^f^Adjusted odds ratio for vaccination status using multivariate logistic regression analysis.

^g^Derived from Fisher exact test.

^h^Five women living with HIV; 1 HIV-uninfected woman.

^i^One hundred thirty-five women living with HIV; 144 HIV-uninfected women.

^j^Four women living with HIV; 2 HIV-uninfected women.

^k^One hundred thirty-six women living with HIV; 143 HIV-uninfected women.

### Association of Strain-specific HAI Titers and Homotypic Influenza Illness

The homotypic HAI GMT (1 month postvaccination in IIV3 recipients and baseline in placebo recipients) was lower in women who developed A/H1N1 illness compared with those who did not (15.5 vs 49.2, respectively; *P* = .02). Furthermore, women developing A/H1N1 illness were 84% (OR, 0.16; *P* = .01) less likely than those without A/H1N1 illness to have A/H1N1 HAI titers ≥40 (18.1% vs 57.2%, respectively) (*P* = .01; Table 5 and Supplementary Figure 7). The HAI titers to A/H1N1 were associated with significantly lower odds of A/H1N1 illness (OR, 0.30; *P* = .03), however, this was not significant after adjusting for HIV (aOR, 0.41; *P* = .10) or vaccine status (aOR, 0.32; *P* = .07). No difference was observed in homotypic HAI GMT in women with and without illness due to A/H3N2 (20 vs 29.8, respectively; *P* = .43) or B/Victoria (14.1 vs 41.6, respectively; *P* = .34). Furthermore, the percentage of women with HAI titers ≥40 to the homotypic virus was not different between those with and without illness due to A/H3N2 (33.3% vs 41.5%, respectively; *P* > .99). None of the women with B/Victoria illness compared to 46.2% who did not develop illness had B/Victoria HAI titers ≥40, albeit not significant (*P* = .50).

**Table 5.** Hemagglutination Inhibition Titers and Association With Confirmed Influenza Illness

All IIV3- or Placebo-Vaccinated Women	A/H1N1 Illness (n = 11)^a^	A/H1N1 Illness (n = 274)^b^	*P* Value^c^	OR (95% CI); *P* Value^d^	aOR^e^ (95% CI); *P* Value^d^	aOR^f^ (95% CI); *P* Value^d^
A/H1N1 GMT (95% CI)	15.5 (7.4–32.2)	49.2 (40.4–59.9)	.02	0.30 (.10–.90); .03	0.41 (.13–1.21); .10	0.32 (.09–1.12); .07
A/H1N1 HAI titer	A/H1N1 Illness, Proportion (%)	No A/H1N1 Illness, Proportion (%)	OR (95% CI); *P* Value^g^			
≥40	2/11 (18.1)	157/274 (57.2)	0.16 (.03–.64); .01			
	A/H3N2 Illness (n = 6)^h^	No A/H3N2 Illness (n = 279)^i^	*P* Value^c^			
A/H3N2 GMT (95% CI)	20 (4.0–98.44)	29.8 (25.1–35.41)	0.43			
A/H3N2 HAI titer	A/H3N2 Illness, Proportion (%)	No A/H3N2 Illness, Proportion (%)	OR (95% CI); *P* Value^g^			
≥40	2/6 (33.3)	116/279 (41.5)	0.7 (.1–3.0); >.99			
	B/Victoria Illness (n = 2)^j^	No B/Victoria Illness (n = 283)^k^	*P* Value^c^			
B/Victoria GMT (95% CI)	14.1 (.17–1156)	41.6 (35.0–49.3)	.34			
B/Victoria HAI titer	B/Victoria Illness, Proportion (%)	No B/Victoria Illness, Proportion (%)	OR (95% CI); *P* Value^g^			
≥40	0/2 (0)	131/283 (46.2)	0 (0–2.5); .50			
	B/Victoria or B/Yamagata Illness (n = 6)^l^	No B/Victoria or B/Yamagata Illness (n = 279)^m^	*P* Value^c^	OR (95% CI); *P* Value^d^	aOR^e^ (95% CI); *P* Value^d^	aOR^f^ (95% CI); *P* Value^d^
B/Victoria GMT (95% CI)	14.1 (9.4–21.0)	42.2 (35.5–50.2)	.04	0.15 (.01–1.28); .08	0.16 (.01–1.36); .09	NA
B/Victoria HAI titer	B/Victoria or B/Yamagata Illness, Proportion (%)	No B/Victoria or B/Yamagata Illness, Proportion (%)	OR (95% CI); *P* Value^g^			
≥40	0/6 (0)	131/279 (46.9)	0 (0–.81); .03			

Abbreviations: aOR, adjusted odds ratio; B/Victoria or Yamagata, Influenza B/Yamagata strain; CI, confidence interval; GMT, geometric mean titer; HAI, hemagglutination inhibition; IIV3, trivalent inactivated influenza vaccine; NA, analysis could not be adjusted for vaccination status as all influenza illness cases were in placebo group; OR, odds ratio.

^a^Ten women living with human immunodeficiency virus (HIV); 1 HIV-uninfected woman.

^b^One hundred thirty women living with HIV; 144 HIV-uninfected women.

^c^Derived from Mann-Whitney test.

^d^Derived using logistic regression analysis.

^e^Adjusted odds ratio for HIV status using multivariate logistic regression analysis.

^f^Adjusted odds ratio for vaccination status using multivariate logistic regression analysis.

^g^Derived from Fisher exact test.

^h^Five women living with HIV; 1 HIV-uninfected woman.

^i^One hundred thirty-five women living with HIV; 144 HIV-uninfected women.

^j^One woman living with HIV; 1 HIV-uninfected woman.

^k^One hundred thirty-nine women living with HIV; 144 HIV-uninfected women.

^l^Four women living with HIV; 2 HIV-uninfected women.

^m^One hundred thirty-six women living with HIV; 143 HIV-uninfected women.

### H1/Stalk Antibody and HAI Titers Association With Protection Against Influenza Illness

The H1/stalk IgG and HAI titers showed significant correlation in the composite of IIV3 and placebo recipients for A/H1N1 (*r* = 0.65), A/H3N2 (*r* = 0.46), and B/Victoria (*r* = 0.52) (*P* < .0001 for all comparisons; Supplementary Table 4). In the logistic regression analysis, the association of H1/stalk IgG and lower odds for A/H1N1 illness remained significant (aOR = 0.09; *P* = .02) after adjusting for HAI titers (Table 6). The A/H1N1 HAI titer association with lower odds of A/H1N1 illness was not significant after adjusting for H1/stalk IgG concentrations (aOR, 0.71; *P* = .62). Also, H1/stalk IgG or B/Victoria HAI titer association with protection against influenza B illness was not significant after adjusting for the other as covariates (Table 7). Analysis limited to women living with HIV also showed H1/stalk IgG being associated with lower odds for A/H1N1 influenza illness (aOR, 0.25 [95% CI, .02–2.18]; *P* = .21), more so than for A/H1N1 HAI titers (aOR, 0.75 [95% CI, .19–2.93]; *P* = .68). However, neither of these associations was significant, most likely due to reduced study power for these analyses (Supplementary Table 6).

**Table 6.** Adjusted Association of H1/Stalk Immunoglobulin G or A/H1N1 Hemagglutination Inhibition Titers and A/H1N1 Influenza Illness Among All Women

Antibody	aOR (95% CI)	*P* Value^a^
H1/stalk IgG	0.09 (.01–.71)	.02
A/H1N1 HAI titer	0.71 (.18–2.74)	.62

Abbreviations: aOR, adjusted odds ratio; CI, confidence interval; HAI, hemagglutination inhibition; IgG, immunoglobulin G.

^a^Multivariate logistic regression analysis.

**Table 7.** Adjusted Association of H1/Stalk Immunoglobulin G or B/Victoria Hemagglutination Inhibition Titers and Influenza B (B/Victoria or B/Yamagata) Illness Among All Women

Antibody	aOR (95% CI)	*P* Value^a^
H1/stalk IgG	0.18 (.01–1.93)	.15
B/Victoria HAI titer	0.23 (.02–2.42)	.22

Abbreviations: aOR, adjusted odds ratio; B/Victoria or Yamagata, Influenza B/Yamagata strain; CI, confidence interval; HAI, hemagglutination inhibition; IgG, immunoglobulin G.

^a^Multivariate logistic regression analysis.

## DISCUSSION

This study demonstrated an association between H1/stalk IgG, independent of H1N1 HAI titers, and lower odds of A/H1N1 (group 1 virus) illness. An H1/stalk IgG threshold of ≥215 was associated with 90% lower risk for A/H1N1 illness. Although A/H1N1 HAI titers were also associated with lower odds of A/H1N1 illness, this was no longer significant after adjusting for H1/stalk IgG concentrations. Although the stratified analysis restricted to women living with HIV was similar to that of the composite population in terms of association of H1/stalk IgG, the lack of statistical significance is probably due to reduction in the study power for such an analysis. Nevertheless, as the majority of the A/H1N1 cases (10/11) were in women living with HIV, these data suggest that protection against A/H1N1 illness in the women living with HIV is more likely associated with H1/stalk antibody responses than that mediated by H1N1 HAI antibody.

We also observed an association of H1/stalk IgG and lower odds for non–group 1 viruses (A/H3N2 or influenza B) illness (the majority of cases had HIV infection), and significantly so for influenza B illness. This association was, however, no longer significant in the multivariate logistic regression after adjusting for strain-specific HAI titers. Hence, the causal association of either HAI or H1 stalk antibody in lowering the odds for influenza B illness is equivocal. Nevertheless, 4 of the 6 influenza B cases were due to infection with B/Yamagata lineage virus, which was a mismatch to the B/Victoria lineage strain in the vaccine. Speculatively, H1/stalk IgG may have possibly played a role in conferring cross-protection against illness from the vaccine mismatched circulating influenza B virus [23]. This is supported by studies showing that the HA stalk domain is more conserved than the HA head domain in amino acids and genetic sequence within the same group subtypes as well as within cross-group subtypes [14]. Amino acid homology within group 1: H1 (A/PR/8/34) and H2 (A/Jap/305/57) viruses was shown to be 79% for stalk and 58% for head; within group 1: H1 (A/PR/8/34) and group 2: H3 (A/Aichi/2/68) was 53% for stalk and 35% for head; and within group 1: H1 (A/PR/8/34) and B (B/Lee/40) was 39% for stalk and 24% for head [14]. Furthermore, the stalk region undergoes limited antigenic variation and as a result contains the most conserved epitopes across strains and HA subtypes for antibody recognition [17, 24, 25].

The neutralization breadth of stalk-specific antibodies is typically group-specific [12], wherein group 1 subtype-specific antibodies typically neutralize subtypes among the same group of influenza strains as reported elsewhere [26, 27]. Human monoclonal antibodies (mAbs) against a conserved epitope within group 1 stalk protein have been isolated that typically cross-react within the same group in animal model studies [15, 28], and similarly so for human mAbs specific to group 2 stalk protein [29]. Polyclonal antistalk antibodies induced by H5 or H7 influenza virus vaccines in humans also follow the same group-specificity trend in animal models [30–32]. Association of H1/stalk antibodies and protection from A/H1N1 illness in this study corroborates those in previous studies.

Antibody responses following IIV3 were predominantly elicited toward the immune-dominant HA head domain as compared to the immune-subdominant stalk domain in IIV3 vaccinees, both those HIV uninfected and those living with HIV. This is consistent with previous studies on responses against seasonal influenza virus vaccine [2–4, 30]. The fold increase in H1/stalk IgG 1 month post–IIV3 vaccination was lower in HIV-uninfected women and women living with HIV than that observed for HAI responses. Furthermore, in IIV3 recipients, the H1/stalk and HAI concentration did not correlate following immunization in HIV-uninfected women, but correlated in women living with HIV. This could be due to the higher prevaccination A/H1N1 HAI titers in HIV-uninfected women attenuating the A/H1N1 HAI immune responses [33, 34], more so than in women living with HIV, who had lower prevaccination HAI titers. The strong correlation between the pre- and post-IIV3 vaccination H1/stalk IgG responses suggest that there was less interference to H1/stalk IgG responses in women with HIV and in HIV-uninfected women. It further indicates a strong stalk-specific memory B-cell recall response against the conserved stalk epitopes within naturally circulating strains and vaccine strains. In conclusion, although IIV3 induces modest H1/stalk IgG responses, such responses could be boosted by a H1/stalk vaccine, which could be an important pathway to the development of a universal influenza virus vaccine at least targeted at group 1 influenza virus.

Limitations of our study include it being an exploratory study and not being adequately powered to address the association of H1/stalk and protection against A/H1N1 separately in women living with HIV and HIV-uninfected women.

## Supplementary Data

Supplementary materials are available at *Clinical Infectious Diseases* online. Consisting of data provided by the authors to benefit the reader, the posted materials are not copyedited and are the sole responsibility of the authors, so questions or comments should be addressed to the corresponding author.

ciz927_suppl_Supplementary_Material

## Notes

Notes

***Author contributions.*** S. A. M., N. D., M. C. N., G. K., R. N., and F. K. participated in conception of the study. S. A. M., C. C., and M. C. N. enrolled study participants and collected samples. N. D. performed the laboratory work. F. K. and R. N. provided the H1/stalk protein. N. D., G. K., S. A. M., and A. I. analyzed the data. N. D. and S. A. M. drafted the manuscript. All authors reviewed and edited the manuscript.

***Acknowledgments.*** The authors acknowledge Dr Adriana Weinberg (University of Colorado, Denver) for testing of HAI titers.

***Disclaimer.*** The funders of the study had no role in study design, data collection, data analysis, data interpretation, or writing of the report. The corresponding author had full access to all the data in the study and had final responsibility for the decision to submit for publication.

***Financial support.*** This work was supported by the Bill & Melinda Gates Foundation (BMGF grant number OPP1002747); the Medical Research Council, South Africa; and the Department of Science and Technology–National Research Foundation, South Africa. N. D. was granted postdoctoral research fellowship for this work from the Claude Leon Foundation, South Africa; work in the Krammer laboratory was supported by the BMGF and by the National Institute of Allergy and Infectious Diseases' Centers of Excellence for Influenza Research and Surveillance (HHSN272201400008C).

***Potential conflicts of interest*.** The Icahn School of Medicine at Mount Sinai has filed patent applications regarding influenza virus vaccines naming F. K. and R. N. as inventors. C. C. has received institutional grants from BMGF, Pfizer, and IMPRINT, and consulting fees from Pfizer. S. M. has received institutional grants from BMGF, Pfizer, GSK, Minervax, and Sanofi, and advisory committee fees from BMGF. F. K. has received grants from GlaxoSmithKline, the US Department of Defense, and PATH. M. N. has received travel grants from Sanofi Pasteur. R. N. has contributed to GSK-funded vaccine research work. All other authors report no potential conflicts of interest. All authors have submitted the ICMJE Form for Disclosure of Potential Conflicts of Interest. Conflicts that the editors consider relevant to the content of the manuscript have been disclosed.
