# Supplementary material for: Hemagglutinin Stalk Antibody Responses Following Trivalent Inactivated Influenza Vaccine Immunization of Pregnant Women and Association With Protection From Influenza Virus Illness
Source: Clin Infect Dis. 2019 Sep 27;71(4):1072–9. doi: 10.1093/cid/ciz927 (PMC7428398; doi:10.1093/cid/ciz927)
Supplement: ciz927_suppl_Supplementary_Material [file ciz927_suppl_supplementary_material.docx]

**SUPPLEMENTARY INFORMATION**

**Detection of H1/stalk antibodies**

For detection of H1/stalk IgG antibodies, chimeric H6/1 recombinant protein was utilised containing an H6 head domain (to which humans are naïve) linked with an H1 stalk domain, to which humans are known to have pre-exiting immunity [[1](#_ENREF_1)]. Chimeric H6/1 stabilized stalk protein (H6 head from A/mallard/Sweden/81/02 virus combined with an H1 stalk from A/California/04/09) was expressed in the baculovirus expression system [[2](#_ENREF_2), [3](#_ENREF_3)]. Reactivity against this protein measures antibodies against the H1 stalk domain [[4](#_ENREF_4), [5](#_ENREF_5)].

Plasma samples were tested for IgG using ELISA. Briefly, microtiter plates (Cat: 3855, Thermo Scientific, USA) were coated with recombinant protein diluted to 2 µg/ml in 0.1 M carbonate buffer pH 9.4, overnight at 4ºC. Plates were washed and subsequently blocked with blocking solution [3% fetal bovine serum (Biowest, USA), 0.5% non-fat dry milk powder (Bio-Rad, USA)] for 2h at room temperature (RT). After washing, plasma samples were added to the plate in true duplicates diluted to a starting concentration of 1:40, followed by two-fold serial dilution and incubated for 2h at RT. Following a wash, anti-human IgG (Fab specific)-peroxidase antibody (Sigma, USA) diluted to 1:3000 in blocking solution was added to the plate. After 1h incubation at RT, plates were washed and developed using SigmaFast *o*-phenylenediamine dichloride (OPD) (Sigma, USA) for 10 min at RT. The reaction was stopped by adding 3M HCl and the optical density (OD) was measured at 490 nm. The antibody concentration was quantified against the standard curve included on each plate that consisted of polyvalent human normal immunoglobulin (Polygam) (National Bioproducts Institute, South Africa) with an assigned arbitrary value of 1000 arbitrary units (AU)/ml. High, low, and negative controls were also included on each plate. The results are reported in AU/ml. The lower detection limit of the assay was 1.24 AU/ml. Plasma samples were previously tested for HAI titers at University of Colorado in Denver as described [[6](#_ENREF_6)]

**SUPPLEMENTARY TABLES**

**S-table 1: Baseline demographic and clinical characteristics of HIV-uninfected and HIV-infected women in inactivated influenza vaccine (IIV3) and placebo recipients.**

| **HIV uninfected women** | **IIV3 recipients**  n=68 | **Placebo recipients**  n=77 | **p value** |
| --- | --- | --- | --- |
| Mean age; years ± standard deviation | 26.0 ± 5.3 | 25.8 ± 4.9 | 0.90^##^ |
| Body mass index; median [IQR] **^§^** | 28.5; [26.1, 32.3] | 27.4; [23.7, 31.9] | 0.08^∆^ |
| Mean gestational age at enrolment; weeks ± standard deviation | 26.5 ± 4.6 | 26.2 ± 4.0 | 0.77^##^ |
| Gravidity; median [IQR] | 2; [1, 2.75] | 2; [1, 2] | 0.67^##^ |
| Parity; median [IQR] | 1; [0, 1] | 1; [0, 1] | 0.83^##^ |
| Days between pre- and post- vaccination visit; Median [IQR] | 30; [28, 33] | 31; [28, 34] | 0.18^##^ |
| **HIV infected women** | **IIV3 recipients**  n=72 | **Placebo recipients**  n=68 | **p value** |
| Mean age; years ± standard deviation | 26.9 ± 4.9 | 28.8 ± 5.2 | 0.02^##^ |
| Body mass index; median [IQR] **^§^** | 28.5; [25.9, 33.8] | 27.2; [24.3, 31.7] | 0.10^∆^ |
| Mean gestational age at enrolment; weeks ± standard deviation | 27.7 ± 3.9 | 26.8 ± 3.6 | 0.23^##^ |
| Gravidity; median [IQR] | 2; [2, 3] | 2; [2, 3] | 0.19^∆^ |
| Parity; median [IQR] | 1; [1, 2] | 1; [1, 2] | 0.40^##^ |
| Days between pre- and post- vaccination visit; Median [IQR] | 30; [28, 31] | 30; [28, 32] | 0.74^##^ |

§ Calculations for body mass index (weight in kilograms divided by the square of the height in meters) was based on 95 HIV-uninfected women (43 IIV3 recipients and 52 placebo recipients); 113 HIV-infected women (59 IIV3 recipients and 54 placebo recipients), **^##^** Mann Whitney test, ^∆^ Student t test

**S-table 2: Comparison between baseline demographic and clinical characteristics of participants tested and not tested for H1/stalk antibodies in inactivated influenza vaccine (IIV3) and placebo recipients.**

| **IIV3 recipients** | **Tested participants**  n=140 | **Not tested participants**  n=20 | **p value** |
| --- | --- | --- | --- |
| Mean age; years ± standard deviation | 26.5 ± 5.1 | 28.5 ± 4.0 | 0.06^##^ |
| Body mass index; median [IQR] **^§^** | 28.5; [26.0, 32.9] | 27.9; [25.8, 29.0] | 0.10^∆^ |
| Mean gestational age at enrolment; weeks ± standard deviation | 27.1 ± 4.3 | 26.2 ± 3.9 | 0.38^##^ |
| Gravidity; median [IQR] | 2; [1, 3] | 2; [2, 3] | 0.18^##^ |
| Parity; median [IQR] | 1; [0, 1.75] | 1; [1, 1.75] | 0.44^##^ |
| **Placebo recipients** | **Tested participants**  n=145 | **Not tested participants**  n=17 | **p value** |
| Mean age; years ± standard deviation | 27.2 ± 5.2 | 29.8 ± 5.0 | 0.05^##^ |
| Body mass index; median [IQR] **^§^** | 27.3; [23.9, 31.8] | 28.8; [24.1, 32.0] | 0.65^∆^ |
| Mean gestational age at enrolment; weeks ± standard deviation | 26.5 ± 3.8 | 26.7 ± 3.6 | 0.77^##^ |
| Gravidity; median [IQR] | 2; [2, 3] | 2; [2, 3] | 0.60^##^ |
| Parity; median [IQR] | 1; [0, 2] | 1; [0.5, 2] | 0.70^##^ |
| **Overall** | **Tested participants**  n=285 | **Not tested participants**  n=37 | **p value** |
| Mean age; years ± standard deviation | 26.9 ± 5.2 | 29.1 ± 4.5 | 0.009^##^ |
| Body mass index; median [IQR] **^§^** | 27.9; [24.7, 32.0] | 27.9; [24.5, 30.8] | 0.47^∆^ |
| Mean gestational age at enrolment; weeks ± standard deviation | 26.8 ± 4.1 | 26.4 ± 3.7 | 0.60^##^ |
| Gravidity; median [IQR] | 2; [2, 3] | 2; [2, 3] | 0.19^##^ |
| Parity; median [IQR] | 1; [0, 2] | 1; [1, 2] | 0.41^##^ |

^§^ Calculations for body mass index (weight in kilograms divided by the square of the height in meters) was based on 208 participants tested [102 vaccine recipients and 4 placebo recipients] and 26 not tested for H1/stalk antibodies [14 vaccine recipients and 12 placebo recipients]. **^##^** Mann Whitney test, ^∆^ Student t test

**S-table 3. HAI titers among inactivated influenza vaccine (IIV3) or placebo vaccinated HIV- uninfected* and HIV- infected pregnant women.**

| **HIV uninfected women** | **IIV3** | | | **Placebo** | | | **p value** | | |
| --- | --- | --- | --- | --- | --- | --- | --- | --- | --- |
|  | n=68 | | | n=77 | | |  |  |  |
|  | **A/H1N1** | **A/H3N2** | **B/Victoria** | **A/H1N1** | **A/H3N2** | **B/Victoria** | **A/H1N1** | **A/H3N2** | **B/Victoria** |
| **Baseline titer**  GMT;[95%CI] | 38;  [29.6, 48.7] | 24.2;  [19.5, 30.1] | 20.2;  [17.9, 22.8] | 25.9;  [19.3, 34.8] | 14;  [11.4, 17.2] | 15.5;  [13.3, 18] | 0.02 ^##^ | 0.0003**^∆^** | 0.01**^∆^** |
| **Post vaccination titer**  GMT;[95%CI] | 210.7;  [161.2, 275.3] | 124;  [90.4, 170.1] | 228.6;  [180.5, 289.5] | 35.9;  [25.6, 50.2] | 15.4;  [12.4, 19] | 20.9;  [17.3, 25.2] | <0.0001**^∆^** | <0.0001**^∆^** | <0.0001**^∆^** |
| **p value** ^#^ | <0.0001 | <0.0001 | <0.0001 | 0.0004 | 0.01 | <0.0001 |  | | |
| **Mean fold change**; [95%CI] | 5.5;  [4.1, 7.4] | 5.1;  [3.7, 6.9] | 11.3;  [8.7, 14.6] | 1.3;  [1.1, 1.6] | 1.0;  [1.0, 1.1] | 1.3;  [1.1, 1.5] |  |  |  |

| **HIV infected women** | **IIV3** | | | **Placebo** | | | **p value** ^##^ | | |
| --- | --- | --- | --- | --- | --- | --- | --- | --- | --- |
|  | n=72 | | | n=68 | | |  |  |  |
|  | **A/H1N1** | **A/H3N2** | **B/Victoria** | **A/H1N1** | **A/H3N2** | **B/Victoria** | **A/H1N1** | **A/H3N2** | **B/Victoria** |
| **Baseline titer**  GMT;[95%CI] | 28;  [21.9, 35.7] | 17.3;  [14.3, 20.9] | 18.1;  [16.2, 20.3] | 13.1;  [10.1, 17.1] | 11.6;  [9.8, 13.8] | 14.4;  [12.5, 16.6] | <0.0001^##^ | 0.002**^∆^** | 0.01**^∆^** |
| **Post vaccination titer**  GMT;[95%CI] | 71.9;  [49.2, 105.2] | 40.7;  [29.2, 56.8] | 62.8;  [46.2, 85.5] | 14.5;  [11.0, 19.2] | 13.5;  [11.4, 16.1] | 21.4;  [17.3, 26.5] | <0.0001^##^ | <0.0001**^∆^** | <0.0001**^∆^** |
| **p value**^#^ | <0.0001 | <0.0001 | <0.0001 | 0.04 | 0.01 | <0.0001 |  | | |
| **Mean fold change;** [95%CI] | 2.5;  [1.9, 3.3] | 2.3;  [1.8, 3.0] | 3.4;  [2.5, 4.6] | 1.1;  [0.9, 1.2] | 1.1;  [1.0, 1.2] | 1.4;  [1.2, 1.7] |  |  |  |

IIV3: inactivated influenza vaccine; GMT: geometric mean titer; 95%CI: 95% confidence interval

*Analysis limited to only 2011 participants

**^#^** Wilcoxon matched-pairs signed rank test

**^##^** Mann Whitney test

^∆^ Student t test

Comparison of fold change in A/H1N1 antibody titers between HIV-uninfected and –infected vaccine recipients; p value <0.0001 Mann Whitney test

Comparison of fold change in A/H3N2 antibody titers between HIV-uninfected and –infected vaccine recipients; p value <0.0001 Mann Whitney test

Comparison of fold change in B/Victoria antibody titers between HIV-uninfected and –infected vaccine recipients; p value <0.0001 Mann Whitney test

**S-Table 4: Correlations between H1/stalk IgG concentrations and HAI titers of all IIV3 (post vaccination) or placebo-recipients (baseline).**

|  | | **H1/stalk IgG concentrations vs. A/H1N1 HAI titers** | **H1/stalk IgG concentrations vs. A/H3N2 HAI titers** | **H1/stalk IgG concentrations vs. B/Victoria HAI titers** |
| --- | --- | --- | --- | --- |
| **All IIV3 or placebo vaccinated women**  **n=285** | r [95% CI]  p value | 0.65; [0.57, 0.71]  <0.0001 | 0.46; [0.36, 0.55]  <0.0001 | 0.52; [0.42, 0.60]  <0.0001 |

r: Spearman correlation coefficient; 95%CI: 95% confidence interval

**S Table 5: Correlations between pre- and post- inactivated influenza vaccine (IIV3) vaccination H1/stalk antibody concentrations or HAI titers in HIV- uninfected and HIV- infected women.**

| **IIV3 recipients** | **Pre- vs post- H1/stalk IgG** | **Pre- vs post- A/H1N1 HAI titers** | **Pre- vs post- A/H3N2 HAI titers** | **Pre- vs post- B/Victoria HAI titers** |
| --- | --- | --- | --- | --- |
| **HIV uninfected (n=68)**  r [95%CI], p value | 0.69 [0.54, 0.80], <0.0001 | 0.29 [0.05, 0.50], 0.01 | 0.36 [0.12, 0.55], 0.002 | 0.06 [-0.18, 0.30], 0.59 |
| **HIV infected (n=72)**  r [95%CI], p value | 0.76 [0.64, 0.85], <0.0001 | 0.67 [0.51, 0.78], <0.0001 | 0.70 [0.55, 0.80], <0.0001 | 0.31 [0.07, 0.51], 0.007 |

r: Spearman correlation coefficient; 95%CI: 95% confidence interval

**S-Table 6: Adjusted association of H1/stalk IgG or A/H1N1 HAI titers and A/H1N1 influenza illness among HIV infected women.**

| **Antibody** | **aOR; 95%CI** | **p value^§^** |
| --- | --- | --- |
| H1/stalk antibody | 0.25; [0.02, 2.18] | 0.21 |
| A/H1N1 HAI titers | 0.75; [0.19, 2.93] | 0.68 |

§ Multivariate logistic regression analysis

aOR adjusted odds ratio for H1/stalk IgG and A/H1N1 HAI titers

**SUPPLEMENTARY FIGURES**

**S-figure 1: Consort flow diagram of study participants.**

**
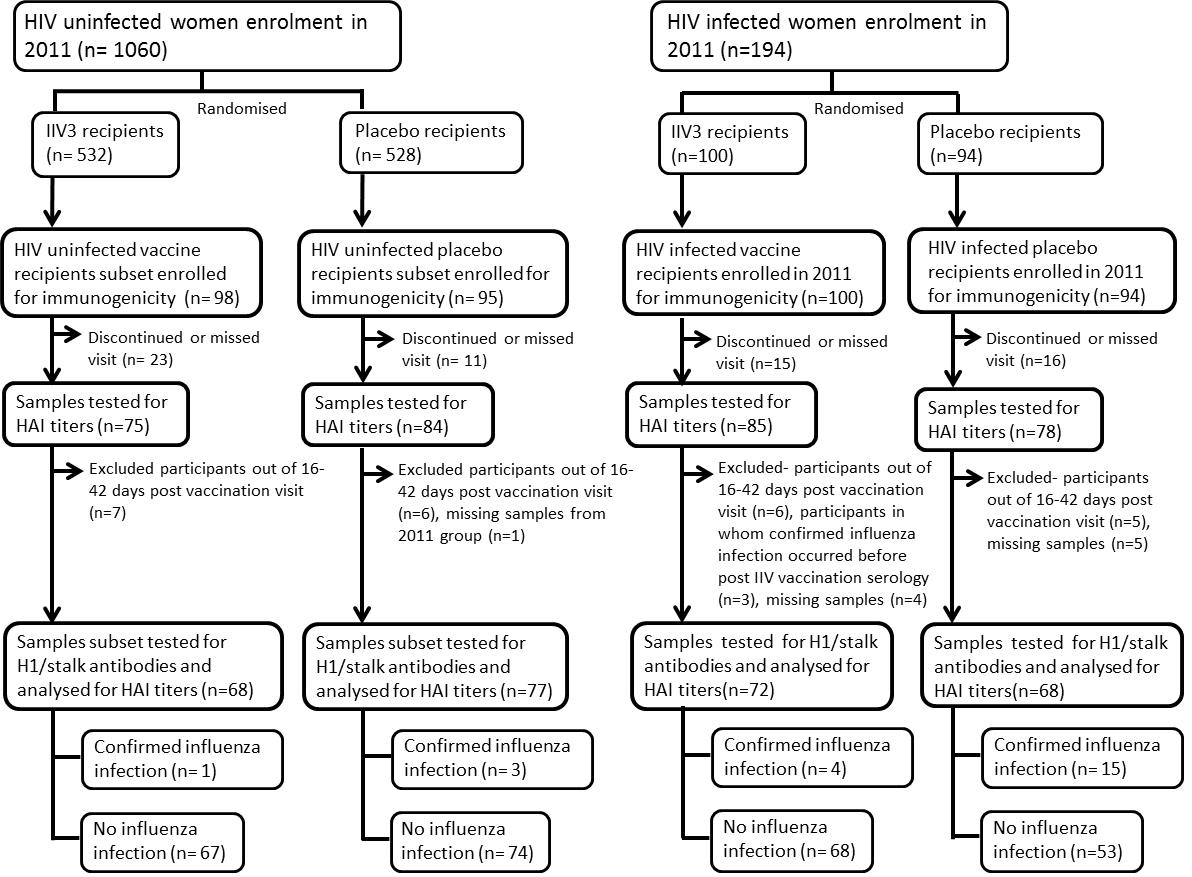
**

**S-figure 2: H1/stalk IgG responses among inactivated influenza vaccine (IIV3) or placebo vaccinated HIV-uninfected and HIV-infected pregnant women.** Represented are median days of sample collection from IIV3 (V) or placebo (P) recipients on X axis, IgG concentrations (black dots) in arbitrary units; AU/ml (in log base 10) on Y axis, GMC (thick solid line), 95%CI (thin solid line), *p values.


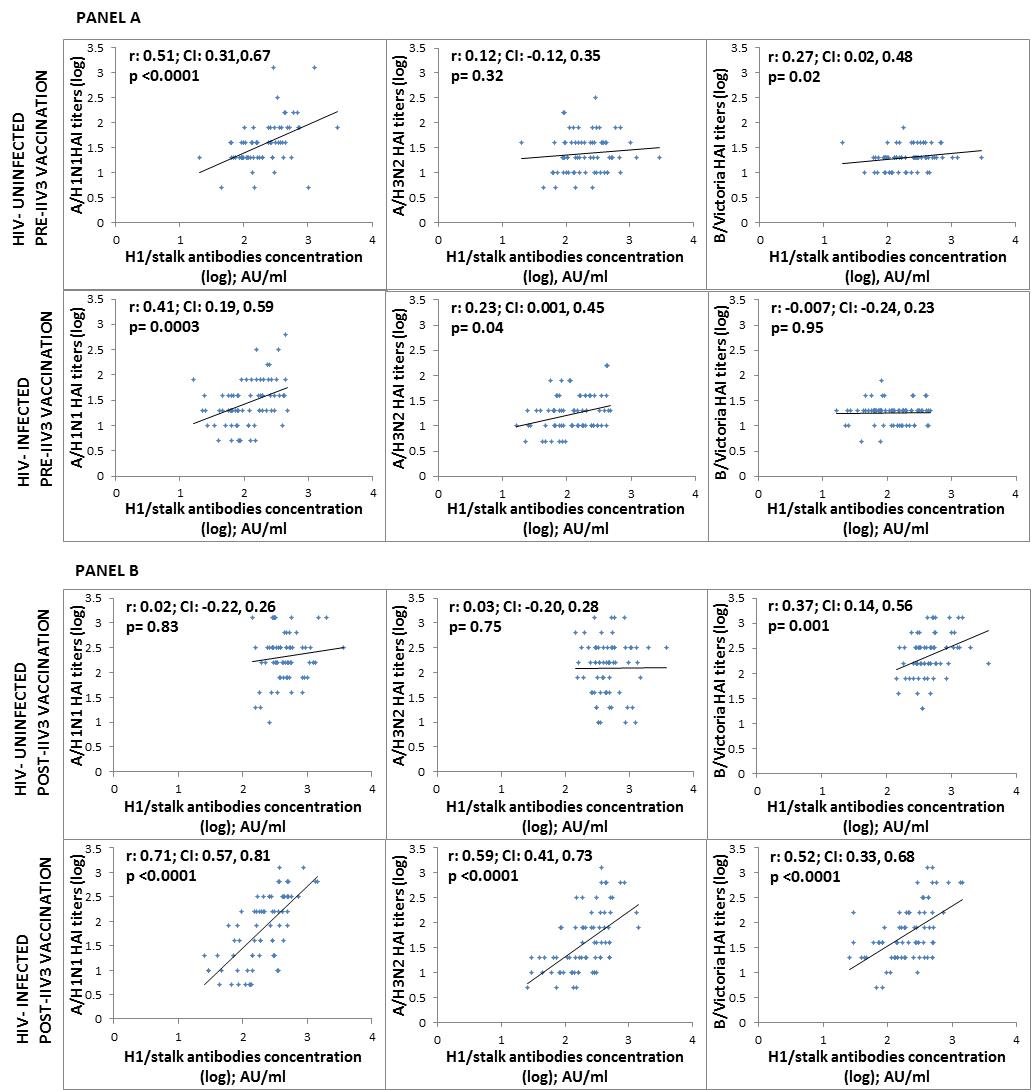


**S-figure 3: Correlations between H1/stalk antibody (IgG) concentrations and HAI titers pre- and post- inactivated influenza vaccine (IIV3) vaccination among HIV-uninfected and HIV–infected women.**


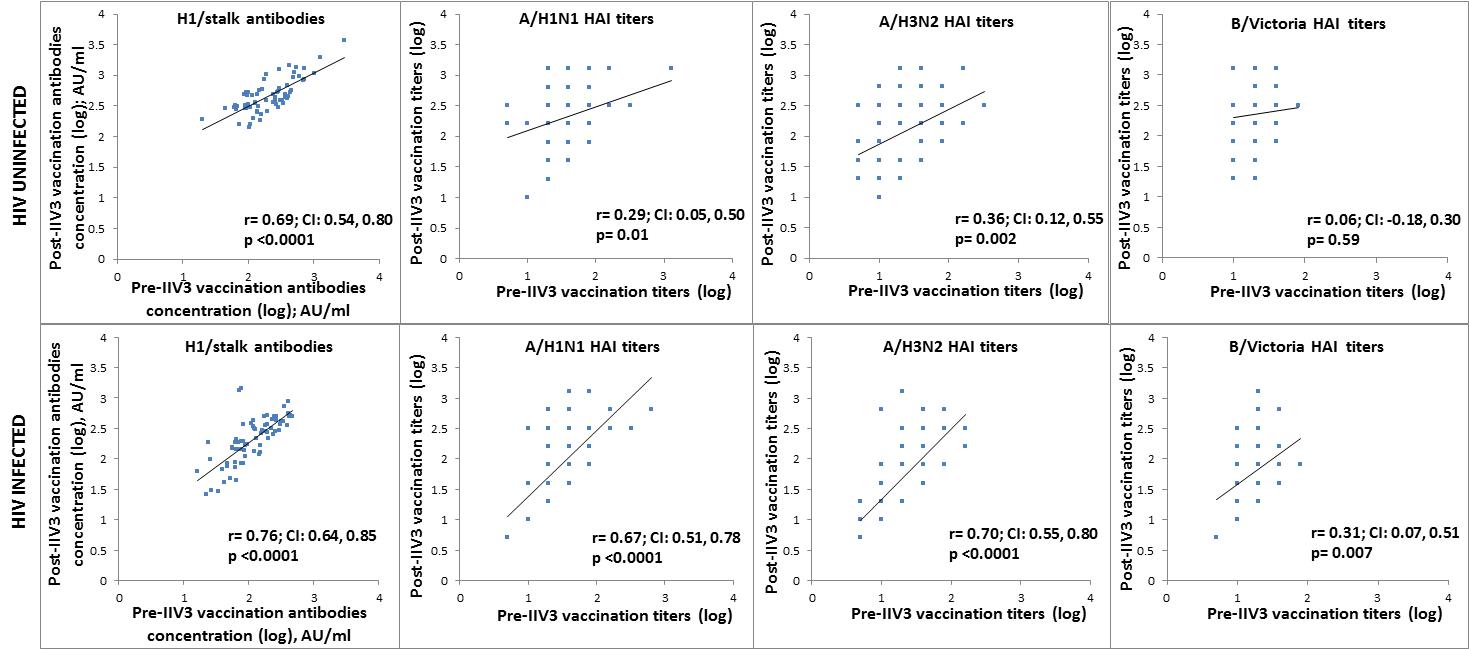


**S-figure 4: Correlations between pre- and post- inactivated influenza vaccine (IIV3) vaccination H1/stalk antibody (IgG) concentrations or HAI titers in HIV- uninfected and HIV- infected women.**

**S-figure 5: Reverse cumulative plots of H1/stalk IgG concentrations of those who developed A/H1N1 influenza-illness and those who remained free of A/H1N1 influenza-illness.** Vertical and horizontal line on X axis and Y axis represent IgG concentration of 215 AU/ml at which 90% women are likely to remain A/H1N1-uninfected.

**S-figure 6: Reverse cumulative plots of H1/stalk IgG concentrations of those who developed non-group 1 (A/H3N2 or B) influenza illness and those who remained free of non-group 1 influenza-illness.** Vertical and horizontal line on X axis and Y axis represent IgG concentration of 330 AU/ml at which 90% women are likely to remain non-group 1-uninfected.

**S-figure 7: Reverse cumulative plots of A/H1N1 HAI titers of those who developed A/H1N1 influenza-illness and those who remained free of A/H1N1 influenza-illness.** Vertical and horizontal line on X axis and Y axis represent A/H1N1-HAI titer equal to 40 at which 84% women are likely to remain A/H1N1-uninfected.

1. Miller MS, Tsibane T, Krammer F, et al. 1976 and 2009 H1N1 influenza virus vaccines boost anti-hemagglutinin stalk antibodies in humans. The Journal of infectious diseases **2013**; 207(1): 98-105.

2. Krammer F, Margine I, Tan GS, Pica N, Krause JC, Palese P. A carboxy-terminal trimerization domain stabilizes conformational epitopes on the stalk domain of soluble recombinant hemagglutinin substrates. PloS one **2012**; 7(8): e43603.

3. Margine I, Palese P, Krammer F. Expression of functional recombinant hemagglutinin and neuraminidase proteins from the novel H7N9 influenza virus using the baculovirus expression system. Journal of visualized experiments : JoVE **2013**; (81): e51112.

4. Nachbagauer R, Wohlbold TJ, Hirsh A, et al. Induction of broadly reactive anti-hemagglutinin stalk antibodies by an H5N1 vaccine in humans. Journal of virology **2014**; 88(22): 13260-8.

5. Nachbagauer R, Choi A, Izikson R, Cox MM, Palese P, Krammer F. Age Dependence and Isotype Specificity of Influenza Virus Hemagglutinin Stalk-Reactive Antibodies in Humans. mBio **2016**; 7(1): e01996-15.

6. Madhi SA, Cutland CL, Kuwanda L, et al. Influenza vaccination of pregnant women and protection of their infants. The New England journal of medicine **2014**; 371(10): 918-31.
